# On the Internal Use of the Nitrate of Silver

**Published:** 1847-11

**Authors:** Richard Southee

**Affiliations:** Cambridge


*On the Internal use of the Nitrate of Silver. By* MR. RICHARD SOUTHEE, *Cambridge.*—The nitrate of silver being much more frequently prescribed than heretofore, it may not be uninteresting to some of your numerous readers to learn that the discolorization of the skin is not of that frequent occurrence generally supposed; in fact, large and repeated doses may be given without producing such effect. In the year 1831, when the cholera raged to a frightful extent at the port of St. Petersburg, I ordered it in grain doses in every case of collapse, and frequently repeated the same quantity every quarter of an hour, without in one instance discolouring the skin. This remedy was mentioned as being very successful, in the report of the Government Commissioners, Drs. Russell and Barry, at the time; and I know of no medicine capable of producing reaction with so much certainty, though this sometimes was sudden and violent, requiring the immediate use of the lancet; and in several cases the inordinate excitement was difficult to suppress, but no discolorization followed. —*London Medical Times.*

